# Controllable Preparation and Research Progress of Photosensitive Antibacterial Complex Hydrogels

**DOI:** 10.3390/gels9070571

**Published:** 2023-07-13

**Authors:** Zhijun Wang, Lili Fu, Dongliang Liu, Dongxu Tang, Kun Liu, Lu Rao, Jinyu Yang, Yi Liu, Yuesheng Li, Huangqin Chen, Xiaojie Yang

**Affiliations:** 1 Hubei Key Laboratory of Radiation Chemistry and Functional Materials, School of Nuclear Technology and Chemistry and Biology, Hubei University of Science and Technology, Xianning 437100, China; qwertasdkl@163.com (Z.W.); fll151852538462022@163.com (L.F.); ldl142325@163.com (D.L.); tdtd800@126.com (D.T.); 17754119632@163.com (K.L.); rl13908440364@163.com (L.R.); yjyxjj@wust.edu.cn (J.Y.); mailyangxiaojie@126.com (X.Y.); 2 Key Laboratory of Coal Conversion and New Carbon Materials of Hubei Province, School of Chemistry and Chemical Engineering, Wuhan University of Science and Technology, Wuhan 430081, China; 3 College of Chemistry and Chemical Engineering, Tiangong University, Tianjin 300387, China; yiliuchem@whu.edu.cn

**Keywords:** controlled preparation, photosensitive antibacterial, hydrogel, application

## Abstract

Hydrogels are materials consisting of a network of hydrophilic polymers. Due to their good biocompatibility and hydrophilicity, they are widely used in biomedicine, food safety, environmental protection, agriculture, and other fields. This paper summarizes the typical complex materials of photocatalysts, photosensitizers, and hydrogels, as week as their antibacterial activities and the basic mechanisms of photothermal and photodynamic effects. In addition, the application of hydrogel-based photoresponsive materials in microbial inactivation is discussed, including the challenges faced in their application. The advantages of photosensitive antibacterial complex hydrogels are highlighted, and their application and research progress in various fields are introduced in detail.

## 1. Introduction

### 1.1. Bacterial Infections and Their Drug Resistance

Bacterial infection is a common problem with the potential to cause significant harm in China and globally [1]. Bacterial infections can cause a variety of diseases, including: (1) respiratory tract infection [2], such as pneumonia and bronchitis, which can lead to respiratory failure in severe cases. (2) Digestive tract infection [3], such as dysentery and cholera, which can lead to dehydration, electrolyte disorders, and other complications in severe cases. (3) Urinary tract infections [4], such as cystitis, pyelonephritis, which can lead to kidney damage in severe cases. (4) Skin and soft tissue infection [5], such as cellulitis and furuncle, which can lead to sepsis in severe cases. (5) Blood infection [6], such as sepsis and septic shock, which is a critical condition with a high fatality rate. Bacterial infections are harmful because they can cause serious illness that deteriorates rapidly, posing a serious threat to the patient’s health. Moreover, bacterial infections can be difficult to treat.

**Figure 1:**
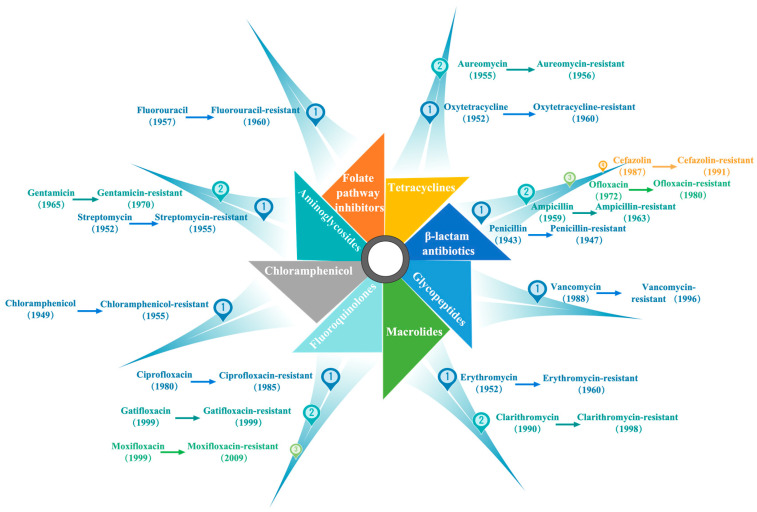
Classification of antibiotics and the history of antibacterial drugs. The left side of the arrowhead is the time when the drug was first marketed, and the right side is the time when the bacteria first developed resistance.

Bacterial infections are also harmful due to an overuse and misuse of antibiotics, causing bacteria to become increasingly resistant to them. This makes it more difficult to treat bacterial infections, which in turn have become one of the world’s biggest public health problems [7,8]. Figure 1 shows the current classification of antibacterial drugs and part of the history of antibacterial drugs. It shows that no matter the type of antibacterial drug, bacteria develop resistance within just a few years. This is because bacteria have a high degree of genetic variation and can produce environmentally adapted mutations within a short period. When bacteria encounter drugs such as antibiotics, the bacteria that are not affected by antibiotics have a greater chance of survival, and they can reproduce and pass on this resistance to future generations of bacteria [9]. As a result, overuse of antibiotics can make it easier for bacteria to develop resistance, which is one of the reasons that antibiotic resistance is growing globally. The emergence of new drug resistance in bacteria has also prompted an urgent search for new, efficient antibacterial materials that are non-toxic, sterile, and biocompatible in contact with humans. To overcome this problem, some novel antimicrobial agents, such as carbon nanotubes [10,11], metal nanoparticles [12,13], polymers [14,15], peptides [16,17], and hydrogels [18,19], have been developed.

### 1.2. Advantages of Antibacterial Hydrogels

Compared with other antimicrobial agents, antibacterial hydrogels have the following advantages: (1) antibacterial hydrogels have low costs and are easy to obtain [20,21,22]. Photosensitive antibacterial complex hydrogels with different antibacterial activities and mechanical strength can be obtained using the method of controllable preparation [23,24,25]. Hydrogels with high antibacterial activity can be obtained by adjusting the process parameters [26]. These hydrogels can avoid resistance by killing the bacteria quickly [27]. (2) Antibacterial hydrogels have a simple method of administration. They have good adhesion for external use and can adhere to the surface of injured organs and tissues [28]. Antibacterial hydrogels also have good injectability for internal use. They can be injected through a needle syringe for minimally invasive treatment of obstructed areas [29,30]. In addition, compared with traditional medical dressings, antibacterial hydrogels can more effectively reduce the risk of wound infection and promote rapid wound healing [31,32]. (3) Hydrogels are excellent carriers. Hydrogels have certain similarities with human tissues in terms of composition, structure, and properties; therefore, they have good biocompatibility and biodegradability, and the release of embedded hydrogels into body fluids can be maintained or controlled [33,34]. Hydrogels are good drug carriers and can reduce the stimulation to the human body [35,36].

### 1.3. Antibacterial Mechanism

#### 1.3.1. Endogenous Sterilization

The endogenous sterilization of materials refers to the ability of a material itself to inhibit or kill bacteria, fungi, viruses, and other microorganisms. This bactericidal effect comes from the chemical or physical properties inside the material. It does not require the intervention of external media, and it is a naturally occurring process [37]. A common internal bactericidal material is Ag [38]. Ag has a broad-spectrum antibacterial effect, allowing it to inhibit the growth of a variety of bacteria and fungi. It also has a killing effect on some viruses. In the fields of medical devices, water treatment, and food packaging, materials containing Ag ions are often used to achieve bactericidal effects. In addition, some natural plant extracts also have endogenous bactericidal effects. For example, tea tree oil and peppermint oil have antibacterial, antifungal, and antiviral effects and can be used in oral care, skin care, and cleaning products [39,40]. In addition, the physical properties of some materials can also achieve internal sterilization. For example, some nanomaterials have a large specific surface area and special surface properties, which can inhibit the growth of bacteria through physical adsorption and charge action [41].

#### 1.3.2. Exogenous Sterilization

Exogenous sterilization refers to the use of external stimuli, such as light [42,43,44,45], magnetic fields [46,47,48], ultrasonic waves [49,50,51], electric fields [52,53,54], microwaves [55,56], and other exogenous antibacterial methods (Table 1) to stimulate materials. Materials can be excited to produce ROS (reactive oxygen species) or heat to achieve the effect of sterilization.

In recent years, microwave spectrum therapy [57], sonodynamic therapy [58], and photoactivation therapy [59] have attracted the attention of researchers as effective and rapid antibacterial methods. Compared with other antibacterial methods, the advantages of exogenous sterilization include convenience, swiftness, strong controllability, a wide application range, a fast reaction speed, environmental protection, energy savings, accurate targeting, and good biocompatibility [60]. This technique can avoid resistance by killing the bacteria quickly without damaging other organs or surrounding tissues [61]. By combining new antibacterial hydrogel materials with photosensitive antibacterial materials, a stronger antibacterial effect can be achieved under the stimulation of exogenous light [62,63], leading to the development of photosensitive antibacterial complex hydrogels. Photosensitive antibacterial complex hydrogels have many advantages. Firstly, they have a broad spectrum of antibacterial activities, which can effectively inhibit the growth of a variety of bacteria, including drug-resistant bacteria. Secondly, they have no toxic side effects on the human body and can be used safely. Finally, due to the water-based matrix of the gel, the material has good biocompatibility and can be widely used in fields such as wound healing and medical device disinfection. Photosensitive antibacterial complex hydrogels have wide application prospects in medical, hygienic, and biotechnology fields. They can be used in the preparation of antibacterial dressings, medical device coatings, and oral care products, which can effectively prevent and treat infectious diseases. In addition, these gels can also be used in areas such as environmental hygiene and food safety to improve hygiene levels and food antibacterial effects. Overall, photosensitive antibacterial complex hydrogels are a new antibacterial material with a wide range of application prospects, and can play important roles in the medical, health, and biotechnology fields.

This review introduces the controllable preparation and research progress of photosensitive antibacterial complex hydrogels composed of photosensitive antibacterial materials combined with hydrogels. Firstly, the classification and technical principles of photosensitive antibacterial complex hydrogels are summarized, including photothermal therapy-based photosensitive antibacterial complex hydrogels, photodynamic therapy-based photosensitive antibacterial complex hydrogels, and photothermal photodynamic synergistic photoantibacterial complex hydrogels. Secondly, the controllable preparation of photosensitive antibacterial complex hydrogels and their antibacterial activities are reviewed, including radiation, chemical crosslinking, and physical crosslinking. Thirdly, the characteristics of the three methods for preparing photosensitive antibacterial complex hydrogels are summarized. Subsequently, the application of photosensitive antibacterial compound hydrogels in biomedicine, food safety, and other fields is introduced. Finally, the existing problems of photosensitive antibacterial complex hydrogels are discussed, and prospective future efforts are proposed.

## 2. Classification and Technical Principles of Photosensitive Antibacterial Complex Hydrogels

According to their mechanism of action, photosensitive antibacterial complex hydrogels can be divided into photothermal therapy-based photosensitive antibacterial complex hydrogels, photodynamic therapy-based photosensitive antibacterial complex hydrogels, and photothermal photodynamic synergistic photoantibacterial complex hydrogels. 

**Figure 2:**
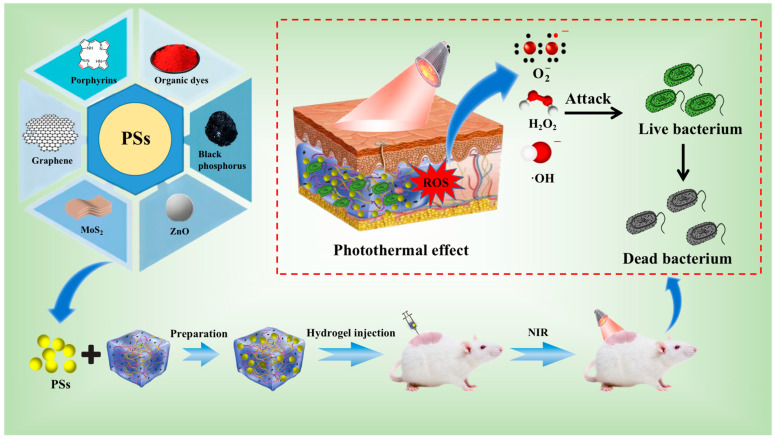
Schematic illustration of the specific mechanism of hydrogels based on PDT.

**Figure 3:**
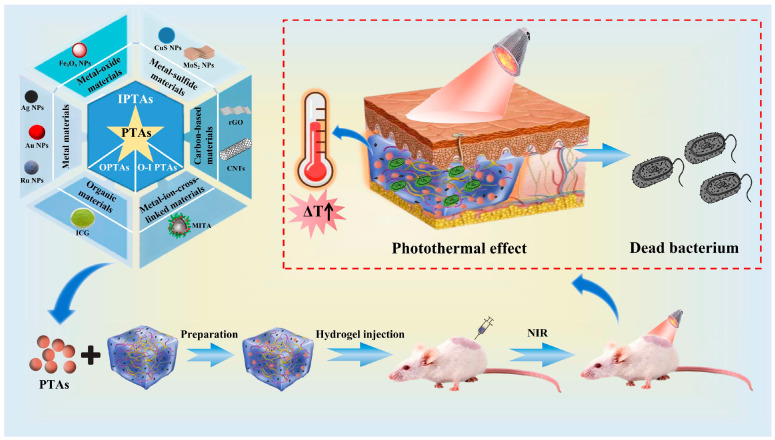
Schematic illustration of the specific mechanism of hydrogels based on PTT.

Figure 2 illustrates the biological mechanism of action of photodynamic therapy (PDT) [64], and Figure 3 shows hyperthermia-based photothermal therapy (PTT) [65]. Both treatments range from ultraviolet (UV) to near-infrared (NIR; NIR is an electromagnetic wave between visible and medium infrared light). A suitable light-activated light-responsive material is selected (usually near-infrared) to quickly and effectively kill bacteria by absorbing light energy to produce reactive oxygen species and/or overheating conditions [66].

### 2.1. Photosensitive Antibacterial Complex Hydrogels Based on PTT

After being stimulated by light, a variety of materials can convert light energy into heat energy. This process leads to the denaturation of internal proteins of bacteria, damage to cell membranes, and ultimately results in direct killing of bacteria. Examples of such materials include precious metal nanomaterials, metal oxides, and polymer nanocomplexes [67,68]. In addition, resistance does not develop without the transmission of genetic information within bacteria, and structural disruption can effectively prevent the formation of biofilms [27,69]. Therefore, this method can directly eliminate the bacteria in the infected part of the wound and promote wound healing. This treatment strategy is known as PTT [70], and the materials used are referred to as photothermal agents (PTAs) [71]. Depending on their source, common PTAs can be divided into three categories: inorganic Ptas (IPTAs) (four categories: metal materials [72,73,74], metal-oxide materials [75], metal–inorganic materials [76,77], and carbon-based materials [78,79]), organic PTA (OPTAs) [80], and organic–inorganic hybrid PTAs (O-I PTAs) [81]. The controllable preparation method of hydrogels combines PTAs and hydrogels, resulting in the preparation of photosensitive antibacterial complex hydrogels based on PTT. These hydrogels can absorb NIR light and generate heat, leading to damage of the bacterial structure through local hyperthermia. This ultimately disrupts membrane permeability and causes bacterial death [82,83]. PTT photosensitive antibacterial complex hydrogels have the advantages of a broad antibacterial spectrum and the absence of bacterial resistance or side effects [84,85].

Despite its effectiveness, Kuo et al. found that PTT alone is sometimes not effective in removing bacteria, and its therapeutic effect varies among patients [86]. Ibelli et al. found that light scattering and absorption effects were unavoidable, thus reducing the efficiency of photothermal conversion [87]. In addition, the thermal ablation temperature of eukaryotic cells exceeded 45 °C, while that of prokaryotic cells exceeded 65 °C. As a result, the temperature was likely to exceed the limits of tolerance of healthy tissue, causing cell damage. This is especially true when fighting drug-resistant bacteria or eliminating biofilms that have formed. The damage to normal tissue caused by direct photothermal therapy is still an urgent problem.

### 2.2. Photosensitive Antibacterial Complex Hydrogels Based on PDT

Controllable preparation of photosensitive antibacterial complex hydrogels based on PDT involves crosslinking or grafting photosensitizers (PSs) with hydrogels. When light of the right wavelength hits the photosensitive antibacterial complex hydrogels, reactive oxygen species (ROS) are produced. Subsequently, a series of photochemical reactions are triggered. Oxidative stress, which occurs when ROS concentrations exceed the limits of a cell’s antioxidant defense system, causes damage to the nucleic acids, proteins, and lipids of bacteria [88]. Currently, commonly used PSs include porphyrins [89], organic dyes [90], conjugated polymers [91], zinc oxide [92], molybdenum disulfide [93], black phosphorus [94], and graphene and its derivatives [95]. PSs produce ROS under light irradiation of the appropriate wavelength to achieve the purpose of killing bacteria. A large number of studies have proved that PSs have an excellent bactericidal effect on both Gram-positive and Gram-negative bacteria [96,97,98]. However, there are also problems, such as low biocompatibility, potential toxicity, low solubility, easy aggregation, and a limited utilization efficiency of visible light [42,99].

### 2.3. PTT and PDT Synergistic Photosensitive Antibacterial Complex Hydrogels

In the photosensitive antibacterial complex hydrogels synergized with PTT and PDT, the combined use of PTT and PDT can realize complementary advantages, which are reflected in the following aspects [100,101,102,103]: (1) Stronger antibacterial effect: PTT is a photothermal agent that converts light energy into heat energy under light stimulation, which can directly kill bacteria by inducing protein denaturation and damaging their cell membranes, resulting in an antibacterial effect. PDT kills bacteria by injecting photosensitizers into them and then photostimulating the reactive oxygen species produced by the photosensitizers. The combined use of the two methods can complement each other and achieve a better sterilization effect. (2) Wider treatment range: PDT and PTT have different treatment ranges. PDT kills microorganisms such as bacteria, viruses, and fungi, while PTT is mainly used to treat diseases such as tumors. Therefore, the combined use of PTT and PDT can complement each other in terms of the therapeutic range, and can be used to treat diseases such as bacterial infections more comprehensively. (3) Reduced usage of photosensitizers. The amount of photosensitizer used is a limiting factor for the application of PDT and PTT. The combination of PTT and PDT can reduce the amount of photosensitizer, thus reducing discomfort and side effects in patients. In conclusion, the combination of PTT and PDT in synergistic photosensitive antibacterial complex hydrogels can realize complementary advantages in terms of antibacterial effect, therapeutic range, and photosensitizer dosage.

## 3. Controllable Preparation of Photosensitive Antibacterial Complex Hydrogels and Their Antibacterial Activity

The preparation methods of photosensitive antibacterial complex hydrogels include radiation crosslinking, chemical crosslinking, and physical crosslinking [104]. The preparation of radiation involves crosslinking photosensitive antibacterial complex hydrogels. The free radicals generated by water radiolysis generate macromolecular free radicals by seizing the hydrogen on the polymer chain, initiating the crosslinking reaction [105,106,107]. The preparation of photosensitive antibacterial complex hydrogels using the physical crosslinking method mainly depends on the physical crosslinking force between molecules. Crosslinks are formed by interactions between non-covalent bonds, such as electrostatic attraction, van der Waals forces, and hydrogen bonds between molecules [108,109,110]. The preparation of photosensitive antibacterial complex hydrogels through chemical crosslinking mainly depends on the forming force of covalent bonds. In the process of preparing hydrogels, covalent bonds are formed between polymer chains through chemical reactions. These reactions cause the polymer chains to be firmly cross-linked together to form a 3D network structure, then the hydrogels are formed [111,112,113]. Table 2 shows the preparation methods, properties, and applications of various photosensitive antibacterial complex hydrogels.

### 3.1. Preparation and Antibacterial Activities of Photosensitive Antibacterial Complex Hydrogels Using Radiation

Radiation sources commonly used for the preparation of hydrogels include ^60^Co [123] and an electron accelerator [124]. γ-rays are extremely penetrating, whereas electron beams are less penetrating [125,126]. The methods of radiation preparation of hydrogels include solid radiation polymerization, aqueous radiation polymerization, and monomer radiation graft copolymerization. Photosensitive antibacterial complex hydrogels are a combination of photosensitive nanoparticles and hydrogels that are formed using radiation technology, which endows hydrogels with remarkable antibacterial properties.

#### 3.1.1. Electron Beam Radiation Preparation

**Figure 4:**
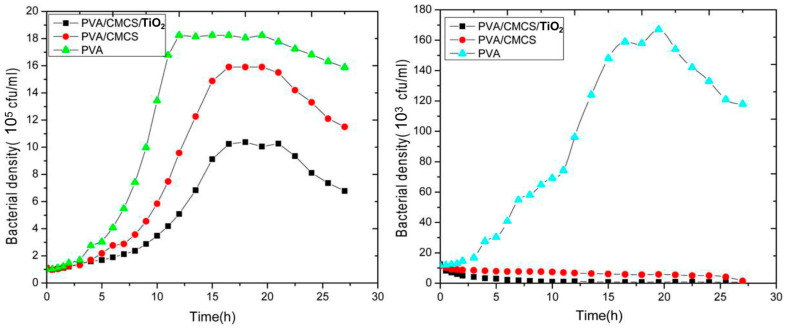
Curves of the bacterial density for different hydrogels against *E. coli* (**left**) and *S. aureus* (**right**). Reprinted with permission from [20].

Li Yuesheng et al. conducted a study using polyvinyl alcohol (PVA), carboxymethyl chitosan (CMCS), and nano-titanium dioxide (TiO_2_) as raw materials. They treated the materials with 30 kGy absorption dose irradiation using physical freeze–thaw and an electron beam with an energy of 1 MeV [20]. Nano-TiO_2_/CMCS/PVA ternary photosensitive antibacterial complex hydrogels were prepared. The antibacterial activity and cytotoxicity of the complex hydrogels were determined using the antibacterial ring method, plate counting method, and cell density method. Figure 4 demonstrates the hydrogel’s significant antibacterial activity against both *Escherichia coli* (*E. coli*) and *Staphylococcus aureus* (*S. aureus*). The synergistic effect between nano-TiO_2_ and the polymer is helpful for improving the antibacterial performance. For *E. coli*, the antibacterial effect of the PVA/CMCS/TiO_2_ photosensitive antibacterial complex hydrogels decreased from the highest concentration of bacteria in PVA hydrogels, 1.8 × 10^6^ cfu/mL (colony forming units), to 1 × 10^6^ cfu/mL. For *S. aureus*, the antibacterial effect of the PVA/CMCS/TiO_2_ photosensitive antibacterial complex hydrogel decreased from the highest concentration of bacteria in the PVA hydrogel of 1.6 × 10^5^ cfu/mL to almost 0. Moreover, the mechanical properties of hydrogels can be accurately regulated by controlling the polymer components and irradiation conditions, which can give hydrogels better water absorption, flexibility, biocompatibilities, effectiveness, and safety. When complex hydrogels are used as photocatalytic agents, the hydrogels can provide an extremely favorable photosensitive synergistic catalytic platform for photocatalysis, which further enhances their antibacterial effects. The hydrogel is made of biodegradable natural polysaccharide material. While the material is slowly degraded, the retained nano-TiO_2_ can be recycled, which achieves the purpose of recycling and further saves on production costs. The combination of photosensitivity and antibacterial properties can change the mechanical properties, physiological properties, biochemical properties, and service life of hydrogels. The synergistic enhancement of the multicomponent complex also changes the photocatalytic pathway, maximizing the effect of nano-TiO_2_.

**Figure 5:**
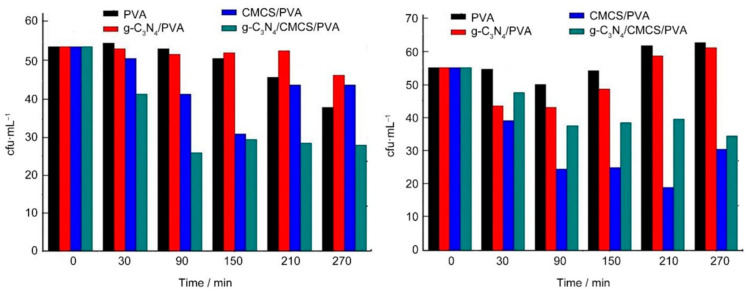
Effects of g-C_3_N_4_/CMCS/PVA on *E. coli* and *S. aureus*. Reprinted with permission from [114].

Li Tingting et al. also prepared carbon nitride (g-C_3_N_4_)/CMCS/PVA ternary photosensitive antibacterial complex hydrogels using cyclic freeze–thaw and electron beam radiation (absorbed dose 30 kGy) [114]. The antibacterial activity of g-C_3_N_4_/CMCS/PVA hydrogels against *E. coli* was superior to that of single-component PVA hydrogels. The CMCS/PVA hydrogels were measured using the antibacterial zone method and plate counting method (Figure 5). The results showed that g-C_3_N_4_/CMCS/PVA photosensitive antibacterial complex hydrogels had excellent antibacterial activity against *E. coli*. However, the disadvantage of these hydrogels is that the antibacterial activity against the Gram-positive bacteria *S. aureus* is low, similar to that of pure PVA hydrogels, and its antibacterial spectrum needs further improvement in the later stage.

Liu Guo et al. used an electronic device with an energy of 1 MeV to carry out radiation treatment of hydrogels and photothermal agents, resulting in a total absorbed dose of 50 kGy [115]. An N-isopropyl acrylamide/highly substituted hydroxypropyl cellulose/ferric oxide (NIPAAm/HHPC/Fe_3_O_4_) complex hydrogel with a pH/temperature/magnetic synergistic response was prepared. The hydrogel contained Fe_3_O_4_ as a doped photothermal agent that interacted with a magnetic field. This complex hydrogel not only has a great application prospect in controlled release and drug delivery systems, but also has a good antibacterial effect on *E. coli* and *S. aureus*, which is expected to be applied in the field of skin trauma.

Arab et al. dissolved 3.5 g of PVA in 90 mL of distilled water at 90 °C. Subsequently, 1 g of agar was added to the PVA solution and stirred for 1 h. The photosensitizer, zinc oxide nanoparticles (ZnO) with different weight ratios (0.05%, 0.1%, 0.2%), was added to the solution [116]. The solution was placed in an ultrasonic bath at 80 °C for 20 min to remove bubbles, then poured into a mold. Polyvinyl alcohol (PVA)/AGAR/ZnO hydrogels were prepared using a 10 MeV accelerator with a total absorbed dose of 25 kGy. The antibacterial experiments showed that ZnO nanoparticles with different mass ratios had no significant difference in antibacterial action on *Bacillus subtilis*. An analysis of its mechanical properties showed that 0.2% ZnO nanoparticles had the best mechanical properties, and the elongation could reach 140%. The hydrogels had enough strength to resist tearing and are expected to be applied in wound dressings.

#### 3.1.2. γ-ray Radiation Preparation

**Figure 6:**
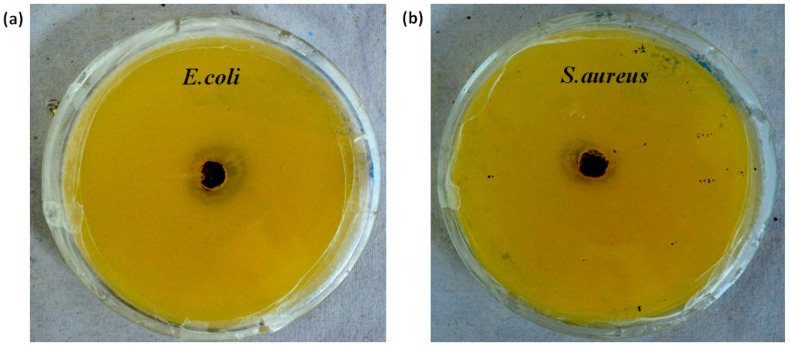
Antibacterial activity screening images of Ag/PVA hydrogel against (**a**) *E. coli* and (**b**) *S. aureus*. Reprinted with permission from [117].

Swaroop et al. used γ-rays for radiation crosslinking of PVA and silver nitrate (AgNO_3_). Ag^+^ was reduced into AgNPs, which were coated with a polyethylene (PVA) matrix [117]. The results showed that Ag/PVA hydrogels showed obvious antibacterial activity against *E. coli* and *S. aureus*, but pure PVA hydrogels showed no antibacterial activity against either bacteria (Figure 6). Swaroop et al. prepared photosensitive zinc oxide (ZnO) and PVA complex hydrogels using γ-rays radiation and studied the antibacterial activity of the complex hydrogels in vitro [118]. The results showed that ZnO/PVA hydrogels had a good killing effect on both Gram-positive and Gram-negative bacteria. Their antibacterial effects might be due to the direct interaction or electrostatic interaction between zinc oxide and the cell surface.

Leawhiran et al. mixed gelatin solutions with PVA solutions of different weight ratios of 100:0, 80:20, and 60:40, respectively, and irradiated them with γ-rays of 30 kGy, 40 kGy, and 50 kGy [119]. The physical performance test showed that when the absorbed dose of irradiation was 30 kGy, the effect of the hydrogel with a mass-to-mass ratio of 60:40 was the best, and the addition of PVA could improve the durability and mechanical integrity. When 0.25%, 0.50%, 0.75%, or 1.00% (according to the solid content) of AgNO_3_ was added, after γ-rays irradiation, AgNPs were formed, which improved the antibacterial performance of the complex hydrogels. Antibacterial experiments showed that when the AgNP content was 1.00%, the antibacterial effect was the best. The characterization of the physical properties, cytotoxicity, and antibacterial activity of the AgNP/gelatin/PVA hydrogels showed that they had appropriate physical properties, non-cytotoxicity, could inhibit the growth of measured bacteria, and could be used as an antibacterial wound dressing.

Mohdy et al. initially prepared 6-chlorobenzo[d]oxazol-2(3H)-one and phosphorus oxychloride, resulting in 6-chloro-2-oxobenzo[d]oxazol-3(2H)-ylphosphonic dichloride [120]. Subsequently, the PVA prepared by crosslinking with a ^60^Co source was stirred and dissolved in a DMF solution. After the solvent was removed, the P-PVA hydrogel was obtained through vacuum drying. Firstly, the photosensitivity of the P-PVA hydrogel was studied using ultraviolet spectroscopy. Secondly, the antibacterial activity of the P-PVA hydrogel against different fungal and bacterial strains was tested using the bacteriostatic zone method. The fungal strains included *Aspergillus fumigatum*, *Aspergillus albicans*, and *Diplocephalus racemosus*, while the bacterial strains included *S. aureus*, *Bacillus subtilis* (as gram-positive bacteria), *Pseudomonas aeruginosa*, and *E. coli* (as Gram-negative bacteria). The results showed that the P-PVA hydrogels had higher activity against fungi and bacteria than PVA hydrogels.

### 3.2. Preparation and Antibacterial Activity of Photosensitive Antibacterial Complex Hydrogels through Chemical Crosslinking

**Figure 7:**
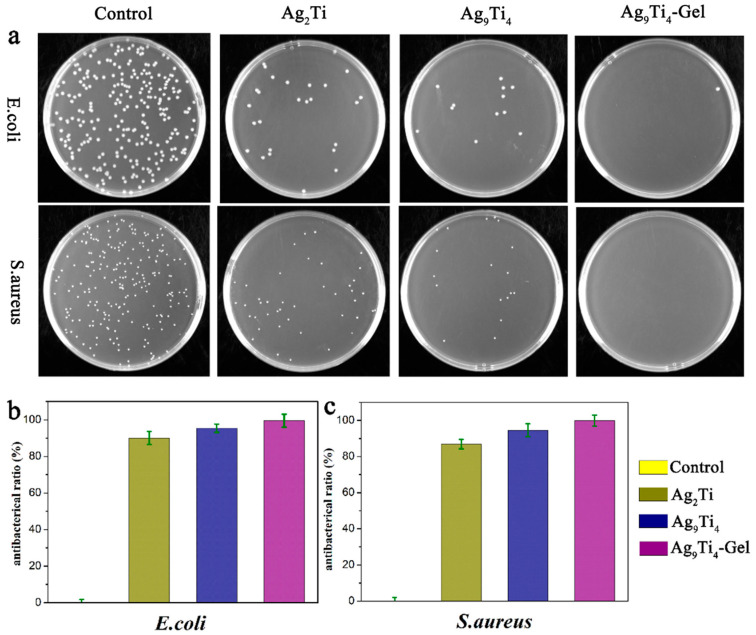
Antibacterial activity of the Ag_2_Ti and Ag_9_Ti_4_ crystals and the Ag_9_Ti_4_-Gel group: (**a**) colonization, (**b**,**c**) quantified antimicrobial efficiencies of *S. aureus* and *E. coli* after treatments with PBS, Ag_2_Ti, Ag_9_Ti_4_, and Ag_9_Ti_4_-Gel. Source: open access [121].

Luo et al. used [Ag(CH_3_CN)_3_][Ag_8_Ti_4_(SA)_12_(CH_3_CN)_2_] (Ag_9_Ti_4_) (SA = salicylic dianion), Ti(O^i^Pr)_4_, salicylic acid, Polyvinyl alcohol (PVA), and Dopamine hydrochloride (DA) to prepare Ag-TOC (Ag_9_Ti_4_-Gel) hydrogels using the one-step solvothermal method [121]. Subsequently, the plate method was used to measure the effectiveness of the samples on Gram-positive (*E. coli*) and Gram-negative bacteria (*S. aureus*). The results showed that the antibacterial rate of Ag_9_Ti_4_-Gel against *S. aureus* and *E. coli* was higher than that of Ag_9_Ti_4_, which further indicated that the Ag_9_Ti_4_ hydrogels had better antibacterial effects (Figure 7). To evaluate the application of Ag_9_Ti_4_-Gel in the photothermal field, a mouse wound model was established. After 12 days, the Ag_9_Ti_4_-Gel + NIR group was the first to heal, and the wound area was smaller than that of the Ag_9_Ti_4_-Gel and Ag_9_Ti_4_ groups. The results showed that the photothermal effect of Ag_9_Ti_4_-Gel can effectively improve the antibacterial activity of the prepared hydrogel. In addition, hematoxylin–eosin (H&E) staining was used to analyze the wound contraction and epithelial cell conditions. The test results indicated that the Ag_9_Ti_4_-Gel + NIR group had faster wound contractions and the best wound healing effects under NIR exposure. Furthermore, cytokines were selected as indicators for the study. The results indicated that the process of wound healing mediated by Ag_9_Ti_4_-Gel under NIR exposure may have been caused by the anti-inflammatory environment provided by Ag_9_Ti_4_-Gel, which greatly increased the concentration of cells related to angiogenesis during skin formation, thus leading to an increase in the number of blood vessels.

Huang et al. initially prepared polyformaldehyde nanoparticles using the one-step oxidation method [101]. Mo_2_C was dispersed into deionized water, a H_2_O_2_ solution was added, and the resulting solution was centrifuged. The supernatant liquid, containing POM nanoparticles, was frozen and dried. In the next step, the POM nanoparticles were dispersed into deionized water, potassium nitrate and a silver nitrate solution were added, and the KCl solution and stirred overnight. Dark blue AgPOM nanoparticles were obtained by freeze-drying the solution after 24 h of dialysis. Finally, the injectable hydrogel was synthesized. Gelatin was dissolved in deionized water, then tea polyphenols (TPs) and urea were added to obtain a T-G-U gel. AgPOM nanoparticles were then added to the resulting T-G-U gel to create an injectable tissue adhesive hydrogel for photothermal/chemodynamic synergistic antibacterial and wound healing promotion. Firstly, AgPOM nanoparticles were incubated with MRSA to evaluate their antibacterial properties in vitro. The results showed that approximately 40% of the MRSA under laser irradiation was killed, while approximately 70% of the MRSA under H_2_O_2_ irradiation was killed, indicating that the ROS produced using AgPOM and H_2_O_2_ significantly enhanced the bactericidal effect. Combined with the photothermal effect of AgPOM, it was able to kill nearly 90% of the bacteria. Then, the prepared hydrogel was used as a wound dressing to observe its antibacterial effect and promote wound healing. The results showed that after three days of near-infrared irradiation, the wound healing rate of the gel group was the highest, exceeding 50% and significantly surpassing the other three groups. In summary, a hydrogel for the synergistic photothermal/chemical kinetic treatment of bacterial infection and to promote wound healing was successfully synthesized and quantitatively evaluated.

Wang et al. used carboxymethyl cellulose (CMC), hydroxypropyl trimethyl ammonium chloride chitosan (HACC), curcumin, and CuS nanospheres prepared via the solvothermal method as raw materials to successfully prepare a biodegradable and self-healing photocontrolled antibacterial hydrogel containing CuS@C nanospheres based on CMCBA and HACC [32]. First, in vitro and in-animal antibacterial experiments were performed on cultures of *E. coli* (ATCC 10536) and *S. aureus* (ATCC25923). The results showed that the CuS@C photosensitive antibacterial complex hydrogel had the highest antibacterial properties against *E. coli* and *S. aureus* under 808 nm near-infrared laser irradiation. The antibacterial activity of the hydrogel against *E. coli* and *S. aureus* was evaluated using live/dead fluorescence staining. The effect of the hydrogel on the permeability of the bacterial membrane was measured using ONPG (higher cellular permeability can lead to protein leakage and thus bacterial death). The antibiofilm activity of the hydrogel was measured using crystal violet staining. The results also proved the excellent antibacterial activity of the CuS@C photosensitive antibacterial complex hydrogel. Finally, a model of back-infected wounds was established to simulate the process of wound healing of the sample, and the results showed that CuS@C hydrogel had the best antibacterial and wound healing ability in vivo. H&E staining and Jimsa staining, which were used to confirm the antibacterial properties and wound-healing activity of the hydrogels, also showed that the CuS@C hydrogels had an excellent ability to promote wound healing, along with good in vivo biosafety.

### 3.3. Preparation of Photosensitive Antibacterial Complex Hydrogels and Their Antibacterial Activity via Physical Crosslinking

**Figure 8:**
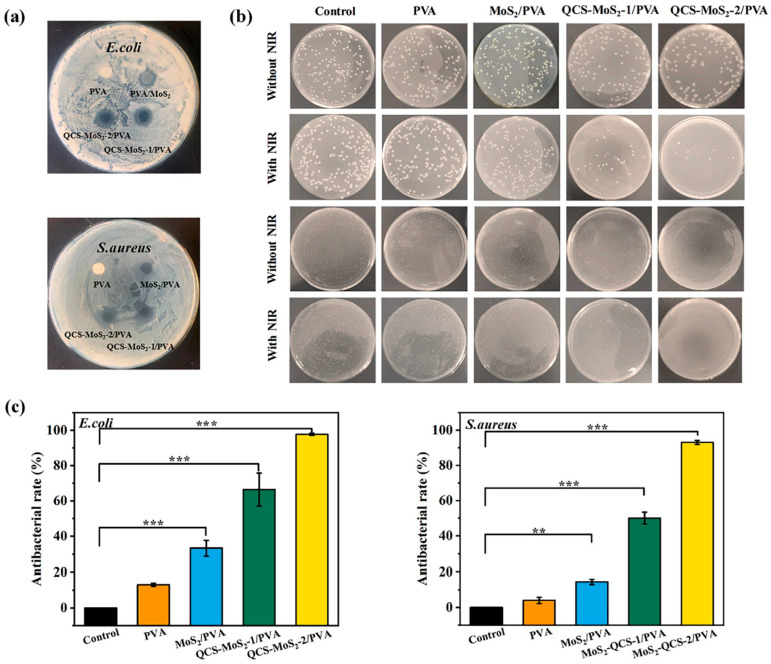
(**a**) Digital images of inhibition zone of the different hydrogels against *E. coli* and *S. aureus*. (**b**) Representative colony forming unit images of *E. coli* and *S. aureus*. (**c**) Antibacterial rate of *E. coli* and *S. aureus* after 808 nm NIR light irradiation for 15 min. (** *p* < 0.01, *** *p* < 0.001). Reprinted with permission from [122].

Yan et al. first synthesized nano-sized molybdenum disulfide using the hydrothermal method, then coated nano-sized MoS_2_ with chitosan quaternary ammonium salt (QCS), and finally added QCs-MOS_2_ to PVA to prepare QCs-MOS_2_/PVA hydrogels via the cyclic freeze–thaw method [122]. QCS-MOS_2_ can be used as an excellent photothermal agent of the near-infrared light response. The antibacterial activity of the QCS-MOS_2_/PVA hydrogel against *E. coli* and *S. aureus* was determined using the bacteriostatic zone method. The results showed that the QCs-MOS_2_/PVA hydrogel had extensive antibacterial activity against *E. coli* and *S. aureus* (Figure 8). Under 808 nm near-infrared light irradiation, the hydrogel had an excellent antibacterial effect. In conclusion, the QCS-MOS_2_/PVA hydrogel is an excellent photosensitive antibacterial compound hydrogel.

Azadikhah et al. first mixed chitosan (CS) dissolved in acetic acid solution with a PVA solution at a ratio of 1:4 to produce a PVA/chitosan solution. Then they added PDI-Ala solution (PDI-Ala as photosensitizer) followed by tannic acid (TA) [34]. A PVA-CS-PDI/TA hydrogel was obtained through freezing and thawing. The antibacterial properties of the hydrogel against *E. coli* and *S. aureus* were measured using the bacteriostatic zone method. The results showed that the PVA-CS-PDI/TA photosensitive antibacterial complex hydrogel had excellent antibacterial properties and could effectively kill bacteria.

## 4. Characteristics of Controllable Preparation of Photosensitive Antibacterial Complex Hydrogels

The characteristics and disadvantages of different hydrogel preparation methods (chemical crosslinking, physical crosslinking, and radiation crosslinking) are shown in Table 3.

### 4.1. Characteristics of Photosensitive Antibacterial Complex Hydrogels Prepared via Chemical Crosslinking

Photosensitive antibacterial complex hydrogels prepared through chemical crosslinking are irreversible once they are prepared, as their interior consists of a three-dimensional network structure formed by covalent bonds [127]. Therefore, the photosensitive antibacterial complex hydrogels prepared using this method are usually stable. However, chemical crosslinking agents (such as catalysts and initiators) are often added to hydrogels prepared via chemical crosslinking [128], and their cytotoxicity and incompatibility with the body greatly affect their biological applications [129].

### 4.2. Characteristics of Photosensitive Antibacterial Complex Hydrogels Prepared via Physical Crosslinking

Photosensitive antibacterial complex hydrogels prepared via physical crosslinking can avoid the use of crosslinking agents that may be cytotoxic, as used in chemical crosslinking methods [130]. Therefore, they have the advantage of good compatibility with biological systems. However, due to the three-dimensional grid structure formed by the non-covalent bond connection between the internal molecules, they are generally reversible, and the solution will be restored when heated [131]. Therefore, the mechanical strength of the photosensitive antibacterial complex hydrogels obtained using this method is poor [132].

### 4.3. Characteristics of Photosensitive Antibacterial Complex Hydrogels Prepared through Radiation Crosslinking

Compared with the above two methods, the photosensitive antibacterial complex hydrogels prepared using the radiation method have the following advantages:

#### 4.3.1. Fast and Efficient

The reason for the rapid and efficient preparation of hydrogels prepared using radiation is that radiation triggers chemical reactions that facilitate the gelation process. Specifically, radiation can trigger a crosslinking reaction of monomers or polymers [133], causing them to form a network structure, thus forming a gel. Hydrogels prepared through the radiation method do not require the addition of any chemical reactants, and only need to be exposed to a monomer or polymer under appropriate radiation conditions. These conditions can quickly form a gel, thus greatly improving the preparation efficiency and shortening the production time. Therefore, the preparation of hydrogels using radiation method has the advantages of being fast and efficient.

Yang et al. prepared an inorganic/organic hybrid poly n-isopropylacrylamide (PNIPAM) hydrogel with polyhedral oligosasiloxane (POSS) using the γ-ray one-step method [134]. Radiation-induced crosslinking is one of the most environmentally friendly, fast, and effective ways to prepare PNIPAM-based hydrogels, as it can be performed without free radical initiators and catalysts.

#### 4.3.2. Extremely Low Cost

The primary reason for the extremely low cost of the method of preparing hydrogels using radiation is because the radiation equipment is a one-time capital investment that can be used multiple times without a large increase in production costs, and the product throughput rate is high. The radiation preparation method for hydrogels can also use conventional raw materials, without the use of expensive catalysts, solvents, and other high-cost raw materials. At the same time, the method is simple to operate and requires less professional equipment, so it can also reduce the cost of preparation.

Alcântara et al. prepared a hydrogel using a simple, elegant, and low-cost ^60^Co source γ-ray process using poly (n-vinyl-2-pyrrolidone) and polyvinyl alcohol (PVA) as the main polymers [135].

#### 4.3.3. Good Biocompatibility

The reason for the good biocompatibility of hydrogels prepared using radiation is that, compared with the chemical crosslinking method, hydrogels prepared using radiation do not need chemical crosslinking agents, do not produce harmful by-products [63], and do not produce organic residue in the preparation process. At the same time, the radiation dose is controlled at an extremely low level, which will not cause too much damage to the properties of the material itself or the tissues and cells of the organism. Since no exogenous chemicals are introduced into the gel prepared using this method, the gel has higher chemical stability and is not prone to decomposition, variation, toxicity, and other problems [136]. Therefore, it is easier to achieve long-term biological applications. In addition, the crosslinking formation of radiation-prepared hydrogels is the formation of covalent bonds between monomer molecules. Compared with chemical crosslinking, the number of crosslinking points is smaller, and the interaction between the crosslinking points is smaller. The water molecules in the gel can be diffused better, making the gel more breathable, transparent, and conducive to tissue growth.

Relleve et al. crosslinked carboxymethyl hyaluronic acid (CMHA) hydrogels using radiation without adding any initiator or crosslinking agent [137]. The CMHA hydrogels prepared under different radiation doses did not show any cytotoxic effects and had good biocompatibility and broad market prospects. Szafulera et al. made glucan-based hydrogels through the coupling of glycide methacrylate with a glucan structure, which was triggered by ionizing radiation [138]. The results of the cytotoxicity evaluation (cell proliferation and cell viability tests), showed that the hydrogel prepared through radiation crosslinking had no cytotoxicity, which indirectly proved that the hydrogel prepared using irradiation in an aqueous solution has a high degree of biocompatibility and has good application in the medical field.

#### 4.3.4. Mild Reaction Conditions and Good Production Controllability

The main reasons for the good controllability of the production of hydrogels prepared using radiation are as follows: (1) The method of preparing hydrogels using radiation is a purely physical method, which can accurately control the radiation dose, irradiation time, irradiation temperature, and other parameters to control the physical and chemical properties of the hydrogels. All these methods can ensure reaction efficiency while reducing the damage to monomer molecules as much as possible. (2) The method of radiation preparation of hydrogels can be prepared by using radiation of different energies (such as gamma rays, electron beams, etc.), so different radiation sources and energies can be selected according to the parameters needed to control the morphology, structure, and properties of the hydrogels [139]. (3) There is no need to add chemical reagents in the preparation process of radiation-prepared hydrogels, which avoids the problems of chemical reaction instability and composition impurities, thus improving the controllability of production. (4) The radiation preparation method for hydrogels can realize continuous production with high efficiency. Meanwhile, the quality of the hydrogels can be monitored and controlled in real-time during the production process, thus improving the production controllability.

Bustamante-Torres et al. proposed a new pH-sensitive hydrogel design that combines acrylic acid (AAc) and AGAR through graft polymerization (gamma ray) copolymerization [140]. The formation of crosslinked hydrogel film was controlled by the radiation intensity and concentration of raw material. It was found that a high radiation dose could improve the degree of crosslinking, and stronger structures could be obtained when the content of the raw material AAc was increased. Ghobashy et al. used dimethylamine ethyl methacrylate/polyoxyethylene oxide (DMAEM/PEO) as the raw material and irradiation crosslinking to obtain a hydrogel film for wound dressing [141]. A (50:50% *v*/*v*) volume ratio and a 20 kGy irradiation dose were used to obtain the best hydrogel film. The above hydrogels with the best performance can be controlled by the production conditions.

#### 4.3.5. Green Environmental Protection and Pollution-Free

Radiation preparation of hydrogels is a green preparation method because it does not require the use of organic solvents and a large number of chemical substances, eliminating the environmental release of volatile organic solvents. This method involves preparing hydrogels by irradiating polymer monomers in aqueous solution. In the process of irradiation, no by-products are generated, and no waste gas, wastewater, and other pollutants are produced [142]. Therefore, it is a very environmentally friendly preparation method. In addition, the physical and chemical properties of hydrogels can be precisely controlled, and the quality and performance of hydrogels can be improved by using the radiation preparation method.

Kanbua et al. successfully prepared Ca^2+^-loaded polyacrylic acid and polyethylene glycol diacrylate (PAA-PEGDA-Ca^2+^) hydrogels using γ-ray irradiation technology [143]. FTIR spectroscopy proved that PAA and PEGDA were successfully cross-linked without byproducts. The whole reaction process has no byproducts, which is a very green and sustainable way to prepare hydrogels.

Overall, compared with the other methods (Table 2) the photosensitive antibacterial complex hydrogels prepared using the radiation method have higher purity, no initiator and catalyst residue, and are more environmentally friendly. Additionally, the produced product has better biocompatibility. Secondly, the production process does not require heating, and the reaction conditions are mild. The crosslinking degree and stability of hydrogels prepared using radiation are higher. Finally, the hydrogel is sterilized using radiation in the radiation synthesis process, which reduces the cost. The irradiation is uniform, the preparation process is simple and convenient for batch preparation, and the preparation cost is lower. Combining the above advantages, the photosensitive antibacterial complex hydrogels prepared using the radiation method are more suitable for industrial scale-up production and applications in daily life.

## 5. Applications

**Figure 9:**
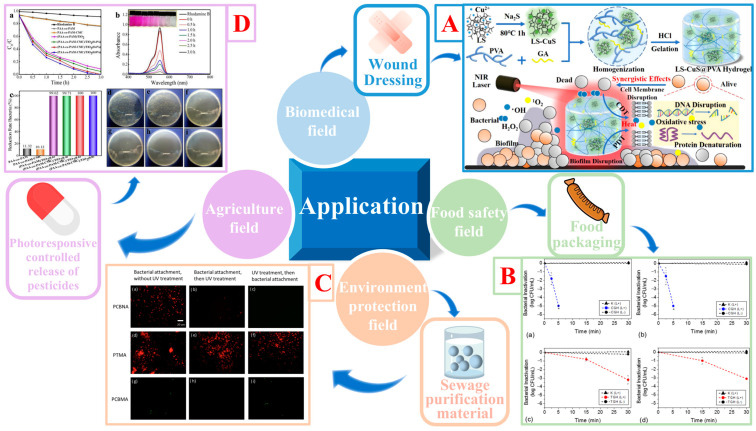
Applications of photosensitive antibacterial hydrogels. (**A**) A LS-CuS@PVA complex hydrogel has been prepared. The hydrogel has near-infrared activated photothermal, photodynamic and peroxide-like properties. Under the conditions of near-infrared (NIR) light irradiation and the presence of H_2_O_2_, the bactericidal effect on *E. coli* and *S. aureus* was significant, and the improvement was attributed to the synergistic effect of high temperature and reactive oxygen species (ROS). Open access [144]. (**B**) Inactivation of *Listeria innocua* incubated on a turmeric–gelatin hydrogel (TGH) or on a cassava–gelatin hydrogel (CGH) and exposed (L+) or not exposed (L−) to UV-A light at 23 °C and 4 °C. (**a**,**b**) represent CGH at 23 °C and at 4 °C, respectively; (**c**,**d**) represent TGH at 23 °C and 4 °C, respectively. The control hydrogel (K) consists of a cassava–gelatin hydrogel without the addition of curcumin. The initial bacterial load was 6 log CFU/mL. The limit of detection was 5 log CFU/mL of bacterial inactivation. Reprinted with permission from [145]. (**C**) Representative fluorescence microscope images of *E. coli* K12 accumulated on polyCBNA (**a**–**c**), polyTMA (**d**–**f**), and polyCBMA (**g**–**i**) hydrogels. The left column (**a**,**d**,**g**) shows bacterial accumulation on the pristine hydrogels without UV treatment. The middle column (**b**,**e**,**h**) shows hydrogels first incubated with bacteria subsequently treated with UV irradiation. The right column (**c**,**f**,**i**) shows hydrogels first treated with UV irradiation then incubated with bacteria. Open access [146]. (**D**) (**a**) Photocatalytic activities for the decomposition of Rh B (1 × 10^−5^ mol·L^−1^, 10 mL) under UV light irradiation. (**b**) Absorption spectral changes of Rh B solution under UV light irradiation in the presence of (PAA-co-PAM-CMC)/TiO_2_(0.6) (inset: photographs of Rh B under UV light irradiation for different time lengths). (**c**) Antibacterial properties of the different hydrogels. Images of bacterial colony distributions of different hydrogels on *E. coli*: (**d**) bare PAA-co-PAM, (**e**) PAA-co-PAM-CMC, (**f**) (PAA-co-PAM)/TiO_2_, and nanocomplex hydrogels with different TiO_2_ contents: (**g**) 0.4, (**h**) 0.6, and (**i**) 0.8 wt%. Reprinted with permission from [147].

Photosensitive antibacterial complex hydrogels represent a new class of materials with photosensitive, antibacterial, and high biocompatibility. Photosensitive antibacterial complex hydrogels are mainly used in the biomedical field, food safety field, environment protection field, and agriculture field (Figure 9).

### 5.1. Biomedical Field

At present, the overuse of antibiotics has led to the development of bacterial resistance, promoting the exploration of excellent biocompatible hydrogels. Historically, photosensitive antibacterial complex hydrogels prepared in a controllable manner were developed in the medical field [148,149], such as in the field of the wound dressing. In the 1990s, hydrogel wound dressings were prepared in China using the radiation crosslinking method. The clinical curative effect showed that hydrogel dressings could play a role in drug release, and long-term use could effectively relieve pain, reduce wound exudation, accelerate wound healing, and reduce the number of dressing changes compared to conventional dressings [150].

Xie et al. successfully prepared a LS-CuS@PVA photosensitive antibacterial complex hydrogel by introducing lignin sulfide copper (LS-CuS) nanocomposites into a polyvinyl alcohol (PVA) hydrogel [144]. The hydrogel had near-infrared activated photothermal, photodynamic, and peroxide-like properties. Through the determination of the photothermal, photokinetic, peroxidase, and antibacterial properties of the LS-CuS@PVA hydrogel, the results showed that the CuS@PVA hydrogel activated using near-infrared could effectively kill bacteria under the synergistic action of photothermal, photodynamic, and peroxidase activities. This work provides a new strategy for treating drug-resistant bacteria. Xu et al. first modified the surface of AgNPs using n-butylamine and oleic acid, then embedded the AgNPs into a calcium alginate (CA) hydrogel and successfully obtained a CA/Ag photosensitive antibacterial complex hydrogel [45]. In vitro, antibacterial tests showed that the CA/Ag hydrogels had photoinduced antibacterial activity against common bacteria and even drug-resistant strains. In vivo, an anti-infection performance test showed that the hydrogel had obvious anti-infection activity in vivo under visible light irradiation. Therefore, the synthesized multifunctional CA/Ag photosensitive antibacterial complex hydrogel is a promising wound dressing. Du et al. first synthesized a photosensitive antibacterial complex hydrogel (PSPG) [151]. Then, in vitro and in vivo experiments showed that the release of PTT, PDT, and NO induced by near-infrared had a synergistic effect on killing bacteria. The proposed photosensitive antibacterial compound hydrogel can effectively kill bacteria and provide a new way to inhibit bacterial resistance.

Professor Li Yuesheng et al. proposed a natural polysaccharide/nano-TiO_2_ complex hydrogel photosensitive antibacterial dressing and radiation synthesis method (ZL 201410313534.X) [152]. The advantages of this invention are that the reaction condition is mild, and the reaction process does not add crosslinking agents, initiators, or any toxic substances to the human body. Additionally, the hydrogels will not have adverse effects on the skin, and they provide of moisturizing and cooling functions as well as hemostasis and astringency functions and bactericidal and bactericidal functions. The hydrogels promote wound healing, absorb wound exudate, and keep the wound environment moist. They are especially suitable for moisture-preserving beauty whitening masks, cooling and antipyretic paste, burns, scalds, and treating sugar urine disease ulcers. The hydrogels can also be used for protection and healing of other wounds, preventing the formation of a scab in the healing process and reducing the formation of scars. Hydrogel preparation, shaping, and sterilization processes can be completed synchronously, greatly simplifying the production process, saving costs, and improve the shelf life and service life of products.

In conclusion, incorporating a breathable backing layer and complex photosensitive antibacterial enhanced hydrogel dressing in direct contact with the skin is an effective way to solve the inherent shortcomings of conventional dressings. This complex hydrogel dressing provides a moist, breathable, and antibacterial environment for the wound, and can fully buffer the impact force of external forces on the wound. These practical advantages of the material present promising real-world application prospects.

### 5.2. Food Safety Field

Photosensitive antibacterial complex hydrogels can be used as natural, low-toxicity, and high-efficiency antibacterial agents in the field of food safety. Their main function is to kill bacteria, fungi, and other microorganisms in food through photosensitization, as well as to extend the shelf life of food and prevent food deterioration. Specifically, photosensitive antibacterial complex hydrogels can be applied to various food preservation, preservative, and disinfection methods, as well as other aspects. For example, in the processing of meat products, a layer of photosensitive antibacterial complex hydrogels can be applied to the surface of meat pieces, and the bacteria on the surface of the meat pieces can be killed using ultraviolet irradiation to achieve the purposes of preservation and preservative.

Juliano V. Tosati et al. first prepared hydrogels with turmeric residue, gelatin or tapioca starch, and gelatin, then added pure curcumin to prepare practical hydrogel coatings with strong antibacterial activity when combined with UV-A light [145]. The coatings were applied to the surface of cooked sausages and evaluated for their ability to prevent Listeria innocua cross-contamination. The results show that the combination of curcumin-supported hydrogel coating with UV-A light had great potential as a photosensitive antibacterial coating to prevent cross-contamination of Listeria innocua in frozen sausages. It should be noted that the application of photosensitive antibacterial complex hydrogels should be carried out in strict accordance with the prescribed concentration and application methods to avoid damage to human health.

At the same time, it is also necessary to pay attention to the treatment of residues and ultraviolet radiation of photosensitive antibacterial complex hydrogels to ensure food safety and environmental protection.

### 5.3. Environment Protection Field

Photosensitive antibacterial complex hydrogels are a new material with a wide range of application prospects. At present, their application in the field of environmental protection is mainly for sewage treatment. Photosensitive antibacterial complex hydrogels can absorb organic matter and heavy metal ions in sewage, and they have antibacterial abilities, which can effectively purify sewage.

Liu et al. first synthesized a photoresponsive polycbNA hydrogel [146]. Cationic hydrogels as precursors can effectively kill attached bacteria, then quickly change into a zionized anti-fouling form through photolysis. This transformation releases the attached bacteria from the surface and prevents further attachment of bacteria. The smart photosensitive CBNA polymer has antibacterial and antifouling properties. Abubshait et al. first prepared PVA/CoZnO NC photosensitive antibacterial complex hydrogels using the coprecipitation method. The results of the antibacterial test showed that the ternary photosensitive antibacterial complex hydrogel had the highest antibacterial activity [153]. The stability of the PVA/CoZnO NC photosensitive antibacterial complex hydrogel to dye photodegradation was verified through recycling experiments. The synthetic photosensitive antibacterial hydrogel can effectively degrade organic pollutants in sewage and can also be used for water disinfection. Mo et al. first synthesized amphiphilic Janus silica particles using the template method, then applied them to the outer surface of hydrogels [154]. Since Janus silica particles contain PDA, the photothermal antibacterial properties of the photosensitive antibacterial hydrogel can be used for antibacterial purposes under light irradiation. This study plays a certain role in the application of hydrogels to environmental antifouling and bacterialization.

### 5.4. Agriculture Field

Photosensitive antibacterial complex hydrogels can play an important role in the field of agricultural control. They are mainly used in the improvement of water quality, as photosensitive antibacterial complex hydrogels can be used as water quality amendments to promote the growth and propagation of soil microorganisms and enhance the resistance of crops to disease. Wang et al. synthesized functional nanocomplex hydrogels using the method of UV-induced free radical polymerization and selected the materials with antifouling function, using acrylamide (AM) and acrylic acid (AA) as the carriers of TiO_2_ NPs [147]. Based on the basket model, these photosensitive antibacterial compound hydrogels have comprehensive photocatalytic, antibacterial, and self-healing functions under sunlight irradiation. The immobilized TiO_2_ NPs provide photoactivity, while the nanocomplex hydrogel matrix offers synergistic antibacterial activity. These photosensitive antibacterial complex hydrogels with comprehensive functions have promising application prospects for maintaining the water quality of crops under solar irradiation.

In addition, they can also be used as sustained release agents of pesticides, and to prolong the action time of pesticides, improve the utilization rate of pesticides, reduce environmental pollution, and improve crop quality. Xing et al. synthesized a new type of polypyrrole@gelatin/poly (acrylic acid) hydrogel with a semi-interpenetrating network structure using gelatin, polypyrrole, and acrylic acid [155]. The photoresponsive release-controlled properties, water absorption properties, and photothermal properties were systematically studied. The results showed that the photosensitive antibacterial complex hydrogels not only had excellent photothermal properties, but also good water retention and photoresponsive pesticide release control performance. These hydrogels have broad application prospects in agricultural applications, and are an effective way to improve pesticide efficiency and reduce environmental pollution.

In conclusion, the application potential of photosensitive antibacterial complex hydrogels in the field of agricultural control is significant, which can provide effective support and guarantee agricultural production.

## 6. Conclusions and Prospects

At present, the methods for controllable preparation of photosensitive antibacterial complex hydrogels primarily include radiation crosslinking, chemical crosslinking, and physical crosslinking. Although chemical crosslinking preparation is stable, the catalyst and initiator can remain in the hydrogel, which has certain biological toxicity. Although the biological toxicity of physical crosslinking preparations is very low, the photosensitive antibacterial complex hydrogels prepared using this method also greatly restrict the practical application of this method due to their instability and poor mechanical strength. Photosensitive antibacterial complex hydrogels prepared using radiation technology have various advantages, including a simple method, a wide range of monomer selection, and a pure product. However, there are some problems with these hydrogels. The photosensitive antibacterial effect needs to be improved, and the interfacial coupling mechanism between inorganic nano-antibacterial particles and hydrogels is still unclear. The photothermal synergistic antibacterial mechanism of inorganic nano-antibacterial particles and hydrogels remains to be further explored. At present, there are still some shortcomings in the research on the technology used to produce hydrogel products in China through radiation crosslinking modulations. Moreover, the initial investment cost of an electron accelerator and cobalt source is significant, and radiation protection measures need to be applied to the workplace. There is still significant room for improvement in the industrial-scale production efficiency of hydrogel products, which also restricts the marketing and promotion progress of new products prepared using radiation to some extent.

The proposed future directions of this work are as follows: (1) Environmental protection: photosensitive antibacterial complex hydrogels can be designed to detect water quality by adsorbing specific pollutants in water and reacting with them. Additionally, these hydrogels can be designed for water purification and pathogen control in aquaculture, reducing pollution to the water environment. They can also be used as a new type of soil remediation agent by adsorbing and degrading contaminated substances to treat and remediate polluted soil. Furthermore, photosensitive antibacterial complex hydrogels can be designed for air purification by adsorbing and decomposing harmful substances in the air, thereby reducing air pollution. (2) Agriculture: These hydrogels can be used for the prevention and control of fruit tree diseases. Photosensitive antibacterial compound hydrogels can be designed as protective agents for the leaves and surfaces of fruit trees to prevent and control fruit tree diseases. Using its antibacterial effect, it can effectively prevent bacterial and fungal infections on the leaves and surfaces of fruit trees, thus improving fruit quality and yield. Moreover, the hydrogels can be designed to be sprayed on crop surfaces to prevent and control crop diseases. For example, in vegetable cultivation, photosensitive antibacterial complex hydrogels can be designed to control leaf vegetable diseases, root vegetable diseases, and fruit vegetable diseases. (3) Personal care: Photosensitive antibacterial complex hydrogels can be used for hand disinfection. By adding them to hand sanitizer and other products, their antibacterial ability is strengthened, effectively preventing hand infections. Additionally, these hydrogels can be added to skin care products, such as creams and masks, to effectively kill bacteria on the skin surface to achieve the effect of skin care. They can also be added to oral care products, such as toothpaste and mouthwash, to effectively prevent problems such as oral infections and cavities. In summary, photosensitive antibacterial complex hydrogels have great potential for application in daily life and production. It is hoped that their controlled preparation, especially those prepared by radiation, can be widely used in these fields.

## Figures and Tables

**Table 1:** Characteristics and antibacterial mechanisms of exogenous photosensitive antibacterial complex hydrogels.

Excitation Source	Characteristics	Mechanisms	Refs
Light	(1) Fast, efficient, and not prone to antibiotic resistance; (2) Green, environmentally friendly, poor tissue penetration depth force, unavoidable shortcomings of light treatment for tissue damage.	(1) Photodynamic therapy: photosensitizers produce cytotoxic ROS under light excitation of a certain wavelength, thus causing oxidative damage to bacteria; (2) Photothermal therapy: photothermic agents generate high temperatures through non-radiative relaxation of electrons excited under light irradiation, resulting in thermal ablation of bacteria.	[42,43,44,45]
Magnetic field	(1) Safe, controllable, good penetration depth of tissue; (2) By using inexpensive, recyclable, and biocompatible superparamagnetic nanoparticles, the intensity and position of the magnetic field can be controlled to achieve targeted sterilization.	(1) Bacteria are captured through electrostatic interactions; (2) Radiation frequency-mediated physical disturbance and bacterial cell membrane dysfunction; (3) Magnetic loss under a magnetic field is converted into heat, and bacteria and biofilms are inactivated by thermal stress.	[46,47,48]
Ultrasonic Wave	(1) Good biocompatibility and safety; (2) Good tissue permeability (>10 cm), and ultrasound energy can be precisely focused on the target, significantly reducing damage to normal surrounding tissues.	(1) Sonodynamic therapy like photodynamic therapy and sonosensitive agents produce ROS under ultrasonic excitation, resulting in oxidative damage; (2) Ultrasonic cavitation can produce shear forces that destroy biofilms and cell membranes.	[49,50,51]
Electric field	(1) High energy utilization efficiency and antibacterial activity; (2) Degradation of electrodes in both electrochemical (direct oxidation or ROS generation) and non-electrochemical (electroporation) processes may result in the release of harmful components.	(1) ROS generation and local electric field enhancement are caused by the unique catalytic activity and physical properties (high conductivity and sharp structure) of the electric field active material; (2) Irreversible electroporation damage caused by a strong electric field to the cell membrane.	[52,53,54]
Microwave	(1) Strong penetration, minor side effects; (2) The energy is much lower than that required to excite any kind of material to induce ROS production.	(1) Excellent thermal conversion efficiency, which can cause thermal ablation of bacteria; (2) Some materials have been proved to mediate the generation of ROS through microwave-induced photodynamics.	[55,56]

**Table 2:** Preparation methods, properties, and applications of various photosensitive antibacterial hydrogels.

Classification	Species of Hydrogels	Materials	Antimicrobial Capability	Applications	Ref.
Radiation crosslinking	Nano TiO_2_/CMCS/PVA ternary photosensitive antibacterial complex hydrogel	Polyvinyl alcohol (PVA), Carboxymethyl Chitosan (CMCS), nano-titanium Dioxide (TiO_2_)	*E. coli*, *S. aureus*	Photosensitive antibacterial	[12]
	g-C_3_N_4_/CMCS/PVA ternary photosensitive antibacterial complex hydrogel	g-C_3_N_4_ (Graphitic carbon nitride), CMCS, PVA	*E. coli*	Photosensitive antibacterial	[114]
	NIPAAm/HHPC/Fe_3_O_4_ complex hydrogel	NIPAAm (N-isopropylacrylamide), HHPC (Hypersubstituted hydroxypropyl cellulose), Fe_3_O_4_	*E. coli*, *S. aureus*	Wound dressing	[115]
	PVA/Agar/ZnO hydrogel	PVA, Agar, ZnO nanoparticles	*B. subtilis bacteria*	Wound dressing	[116]
	Ag/PVA hydrogel	PVA, AgNO_3_	*E. coli*, *S. aureus*	Wound dressing	[117]
	ZnO/PVA hydrogel	ZnO, PVA	*E. coli*, *S. aureus*	Wound dressing	[118]
	AgNP/gelatin/PVA hydrogel	Gelatin, PVA, AgNO_3_	*E. coli*, *S. aureus*, Methicillin-resistant *Staphylococcus aureus* (MRSA)	Wound dressing	[119]
	P-PVA hydrogel	6-chlorobenzo[d]oxazol-2(3H)-one, phosphorus oxychloride, PVA	*Aspergillus fumigatus*, *Geotrichum candidum*, *Candida albicans*, *Syncephal-astrum racemosum*, *Staphylococcus aureus*, *Bacillis subtilis*, *Pseudomonas aeruginosa*, *E. coli*	Drug delivery, Wound healing	[120]
Chemical crosslinking	Ag-TOC hydrogel (Ag_9_Ti_4_ hydrogel)	[Ag(CH_3_CN)_3_][Ag_8_Ti_4_(SA)_12_(CH_3_CN)_2_](Ag_9_Ti_4_),Ti(O^i^Pr)_4_, Salicylic acid, PVA, DA	*E. coli*, *S. aureus*	Treatment of healing wounds	[121]
	AgPOM Multifunctional injectable hydrogel	Gelatin (gel), Tea polyphenol (TP), urea, AgPOM nanoparticles	*S. aureus*, MRSA	Wound dressing	[100]
	CuS@C Photosensitive antibacterial complex hydrogel	carboxymethyl cellulose, hydroxypropyl trimethyl ammonium chloride chitosan (HACC), curcumin, CuS nanospheres	*E. coli*, *S. aureus*	Wound dressing	[32]
Physical crosslinking	QCS-MoS_2_/PVA hydrogel	MoS_2_, chitosan quatenary ammonium salt (QCS), PVA	*E. coli*, *S. aureus*	Biomedical materials, Photothermal antibacterial	[122]
	PVA-CS-PDI/TA hydrogel	Chitosan (CS), PVA, PDI-Ala, tannic acid (TA)	*E.coli*, *S. aureus*	Wound dressing, Cancer treatment	[34]

**Table 3:** Characteristics and disadvantages of different hydrogel preparation methods.

Excitation Source	Characteristics	Disadvantages	Ref.
Chemical crosslinking	A three-dimensional network is formed through cross-linking with covalent bonds, resulting in stable properties and a durable structure.	(1) The catalyst and initiator remain in the hydrogel. The composition of hydrogel is complicated, and the performance of hydrogel is affected; (2) If the initiator or catalyst is toxic, it will further limit the application of hydrogels in the biomedical field.	[127,128,129]
Physical crosslinking	Non-covalent bond forces, such as hydrophobic association forces, hydrogen bonds, electrostatic interactions, coordination bonds, and van der Waals forces, result in cross-linking to obtain a three-dimensional network structure.	(1) Since the chains are reversible and maintain in a steady state, they will recover when heated; (2) Poor mechanical strength.	[130,131,132]
Radiation crosslinking	1. Fast and efficient 2. Extremely low cost 3. Good biocompatibility 4. Mild reaction conditions and good production controllability 5. Green environmental protection and pollution-free Free radicals (·OH, ·H, etc.) generated by water radiation capture hydrogen on the polymer chain to generate macromolecular free radicals, thus triggering cross-linking reactions without adding initiator. The resulting product is pure, with adjustable reaction conditions such as a safe dose and dose rate, high controllability, large range of monomer selection, or it can be directly synthesized from the polymer.	(1) ^60^Co radiation source is extremely radioactive. Improper operation will cause harm to the human body; (2) Electron accelerators are expensive.	[63,133,134,135,136,137,138,139,140,141,142,143]

## Data Availability

No new data were created or analyzed in this study. Data sharing is not applicable to this article.
